# COMPARISON OF TRANSPORTATION USE AMONG OLDER AND YOUNGER PERSONS EXPERIENCING HOMELESSNESS

**DOI:** 10.1093/geroni/igae098.0292

**Published:** 2024-12-31

**Authors:** Sarah Canham, Jeff Rose, Shannon Jones, Ivis Garcia

**Affiliations:** University of Utah, Salt Lake City, Utah, United States; University of Utah, Salt Lake City, Utah, United States; University of Utah, Salt Lake City, Utah, United States; Texas A&M University, College Station, Texas, United States

## Abstract

Regardless of age, transportation determines access to necessary services and supports, including food, healthcare, shelter, and social connection for persons experiencing homelessness (PEH). This study compared experiences and perceptions of transportation among PEH aged <49 years old to PEH aged 50+ years. We conducted a secondary qualitative data analysis of semi-structured interviews with 18 PEH staying in emergency shelters in Salt Lake County (Utah). Using thematic analysis, we identified three points of comparison between older and younger PEH: 1) Modes of transportation used; 2) Reasons for using public transit; and 3) Perceptions of public transit. While there are some similarities across transportation modes used (e.g., public transit, dedicated homeless-services shuttle, walking), older PEH also reported using taxi services when it could be afforded or shared with others. In addition, older PEH more often discussed using public transit to get to healthcare appointments, while younger PEH reported needing transit to get to places of employment. Though both participant groups described the prohibitive costs of transit, OPEH recommended that to use transit most successfully, there need to be places to sit and rest when walking, as well as more publicly available bathrooms and crosswalks for safety. This age-based comparison offers insight into ways to increase transportation equity and support PEH of all ages who similarly have unmet mobility needs.

